# Draft Genome Sequence of Vibrio parahaemolyticus Strain PH1339, Which Causes Acute Hepatopancreatic Necrosis Disease in Shrimp in the Philippines

**DOI:** 10.1128/MRA.01020-19

**Published:** 2019-11-14

**Authors:** Sarah Mae U. Penir, Leobert D. dela Pena, Nikko Alvin R. Cabillon, Angela Denise P. Bilbao, Edgar C. Amar, Cynthia P. Saloma

**Affiliations:** a National Institute of Molecular Biology and Biotechnology, University of the Philippines Diliman, Quezon City, Philippines; b Philippine Genome Center, University of the Philippines, Diliman, Quezon City, Philippines; c Southeast Asian Fisheries Development Center, Aquaculture Department, Tigbauan, Iloilo, Philippines; Georgia Institute of Technology

## Abstract

We report the first draft genome sequence of an acute hepatopancreatic necrosis disease (AHPND)-causing Vibrio parahaemolyticus strain isolated from a Penaeus vannamei sample from the Philippines. The strain carries the genes encoding the Pir-like toxin pair PirA^vp^ and PirB^vp^.

## ANNOUNCEMENT

Acute hepatopancreatic necrosis disease (AHPND), which affects multiple shrimp species, including the economically important Penaeus monodon and Penaeus vannamei, is characterized by massive sloughing of the epithelial cells of the hepatopancreas to the point of atrophy (1). Vibrio parahaemolyticus has been identified as the causative agent of AHPND. In order to mitigate further losses to the shrimp farming industry, it is imperative to identify and characterize the disease-causing strains of V. parahaemolyticus. In the present study, V. parahaemolyticus PH1339 was isolated from a *P. vannamei* sample displaying typical AHPND pathology in a farm in Negros Island, Philippines. Reinfection of *P. vannamei* with the strain replicated the original AHPND pathology.

The hepatopancreas of the AHPND-afflicted *P. vannamei* sample from a farm in Negros Island, Philippines, was dissected and homogenized in sterile seawater for plating in nutrient agar (Pronadisa) with 2% NaCl. The colony of V. parahaemolyticus PH1339 was isolated and grown in nutrient broth (Pronadisa) with 1.5% NaCl incubated overnight at 30°C. Genomic DNA of V. parahaemolyticus PH1339 was extracted from the overnight culture using the KingFisher cell and tissue DNA kit (Thermo Fisher Scientific). A paired-end library was generated from the genomic DNA using the Nextera XT DNA library prep kit (Illumina). The paired-end library was sequenced on the Illumina MiSeq (2 × 300-bp) platform (with the MiSeq reagent kit v. 3) at the DNA Sequencing Core Facility of the Philippine Genome Center at a sequencing coverage of 71×, resulting in a total of 2,784,132 paired-end reads.

The verification of the quality of the raw reads was performed using FastQC (2). The paired-end reads were assembled *de novo* into 57 contigs of sizes greater than 1,000 bp using SPAdes v. 3.13.0 (3) with error and mismatch correction using k-mer sizes of 21, 33, 55, 77, 99, and 127 (*N*_50_, 294,559 bp). Assembly statistics were generated with QUAST v. 5.02 (4). The draft genome assembly of V. parahaemolyticus PH1339 has a length of 5,332,603 bp and a G+C content of 45.2%. PH1339 was assigned as V. parahaemolyticus after the average nucleotide identity (ANI) values between the draft genome of the strain and other publicly available strains of the species were established to be >95% using the aniM option calculate_ani.py (5).

Annotation of the draft genome with Prokka v. 1.13.7 (6) identified 4,819 coding sequences (CDS), 106 tRNAs, 1 transfer-messenger RNA (tmRNA), and 6 rRNAs. The sequence type (ST) of V. parahaemolyticus PH1339 was determined to be ST1166 using the default parameters of SRST2 v. 0.20 (7). Moreover, the plasmid-borne *pirA^vp^* and *pirB^vp^* genes were detected in V. parahaemolyticus PH1339 using SRST2 v. 0.20 (7). The draft genome sequence of PH1339 serves to validate the presence of AHPND in *P. vannamei* farmed in the Philippines and is an additional genomic resource for comparative genomic studies of AHPND strains.

### Data availability.

This whole-genome shotgun project has been deposited at DDBJ/ENA/GenBank under the accession no. VPFJ00000000. The Illumina reads have been deposited under accession no. SRR9937595.
